# Safety analysis of early oral feeding after esophagectomy in patients complicated with diabetes

**DOI:** 10.1186/s13019-021-01410-4

**Published:** 2021-03-26

**Authors:** Zhisheng Jiang, Jing Luo, Mengqing Xu, Zhuangzhuang Cong, Saiguang Ji, Yifei Diao, Yang Xu, Yi Shen

**Affiliations:** 1 Bengbu Medical College, Bengbu, China; 2 Department of Cardiothoracic Surgery, Jinling Hospital, 305 East Zhongshan Road, Nanjing, China; 3 Suzhou Hospital Affiliated To Anhui Medical University, Suzhou, Anhui China

**Keywords:** Esophageal cancer, Type II diabetes, Oral feeding, Nutritional status, Life quality

## Abstract

**Objective:**

To evaluate the safety of early oral feeding in patients with type II diabetes after radical resection of esophageal carcinoma.

**Methods:**

The clinical data of 121 patients with type II diabetes who underwent radical resection of esophageal carcinoma in the department of cardiothoracic surgery of Jinling Hospital from January 2016 to December 2018 were retrospectively analyzed. According to the median time (7 days) of the first oral feeding after surgery, the patients were divided into early oral feeding group (EOF, feeding within 7 days after surgery, 67 cases) and late oral feeding group (LOF, feeding after 7 days, 54 cases). Postoperative blood glucose level, incidence of complications, nutritional and immune indexes, inflammatory indexes, normalized T12-SMA (the postoperative/preoperative ratio of vertical spinal muscle cross-sectional area at the 12th thoracic vertebra level) and QLQ-C30 (Quality Of Life Questionnaire) scores were recorded and compared in the two groups.

**Results:**

There was no statistical difference in preoperative nutritional index and postoperative complication rates between the EOF and LOF group (*p* > 0.05). The postoperative nutritional index (ALB, PA, TRF, Hb) and immune index (IgA, IgG, IgM) of the EOF group were higher than those of the LOF group (*p* < 0.05), and the inflammatory indicators (CRP, IL-6) of the EOF group were significantly lower than those of the LOF group (*p* < 0.05). Moreover, postoperative T12-SMA variation and QLQ-C30 scores of the EOF group were higher than those in LOF group (*p* < 0.05).

**Conclusions:**

Early oral feeding is safe and feasible for patients with type II diabetes after radical resection of esophageal cancer, and it can improve short-term nutritional status and postoperative life quality of the patients.

**Supplementary Information:**

The online version contains supplementary material available at 10.1186/s13019-021-01410-4.

## Introduction

Esophageal cancer is one of the most common digestive malignancies worldwide, with morbidity and mortality rank the 7th and 6th among all malignancies, respectively. GLOBOCAN data showed that 572,000 new cases of esophageal cancer and 509,000 deaths were reported in 2018 over the worldwide [1]. The number of new cases and deaths of esophageal cancer in China ranks the first in the world, accounting for about 50% of the cases and deaths globally [2]. Surgical resection is the main treatment method for patients with esophageal cancer at present. However, the surgical method of esophageal cancer is complex with many postoperative complications, with the 5-year survival rate less than 30% [3].

It has been reported that diabetes may be an independent risk factor for the incidence of esophageal cancer [4]. As a metabolic disorder of glucose, protein and fat caused by defective insulin secretion or impaired biological function, diabetes are growing rapidly all over the world [5]. At present, about 10% ~ 20% of surgical patients are accompanied with diabetes, mainly type II diabetes [6]. For patients of esophageal cancer accompanied with type II diabetes, postoperative complications such as difficult incision healing, pulmonary infection and anastomotic fistula are more likely to occur, which remarkably increases the risk of complications after surgery [7–9]. It is of great significance to reduce the surgical risk of this group of patients.

Nutritional support for patients with esophagectomy is of great significance, especially for patients with malnutrition. However, the timing of oral intake after esophagectomy is still a bone of contention. Based on recent studies, more and more evidences prove that oral feeding is feasible and effective in the early postoperative period of digestive tract surgery [10, 11]. Early oral feeding can reduce the stress response after esophagectomy, accelerate the recovery of intestinal function, and improve the short-term quality of life without increasing postoperative complications and motality [12, 13]. Nevertheless, few researches were reproted to explore the safety of early oral feeding after esophagectomy in patients complicated with type II diabetes. In this study, we compared the incidence of postoperative complications and index of nutrition, immune and inflammation after esophagectomy between early oral feeding group and late oral feeding group in patients complicated with type II diabetes. We aim to investigate the safety and effectiveness of early oral feeding on this kind of patients.

## Materials and methods

### Patients

Retrospective analysis was conducted on the clinical data of patients with esophageal cancer complicated with type II diabetes who underwent radical resection in the cardiothoracic surgery department of Jinling Hospital from January 2016 to December 2018. Inclusion criteria was: (1) all patients were 18 years old or older; (2) esophageal squamous cell carcinoma was diagnosed pathologically after surgery; (3) all patients were diagnosed with type II diabetes as the following creteria:1) Typical diabetes symptoms (thirst, polydipsia, polyuria, polyphagia, unexplained weight loss) and random blood glucose ≥11.1 mmol/ L; 2) Fasting blood glucose FPG ≥ 7.0 mmol/L; 3) Blood glucose at 2 h of OGTT was ≥11.1 mmol/ L; 4) HbA1c ≥ 6.5%; (4) complete clinical data and follow-up information are available. Exclusion criteria was: (1) patients with severe heart disease and liver and kidney dysfunction; (2) severe coagulation dysfunction; (3) concurrent or previous history of other malignant tumors; (4) perioperative death. According to the median time (7 days) of the first oral feeding after surgery, the patients were divided into early oral feeding group (EOF, feeding within 7 days after surgery, 67 cases) and late oral feeding group (LOF, feeding after 7 days, 54 cases). And the patients were staged using the 8th edition of the TNM staging standard for esophageal cancer issued by the Union for International Cancer Control (UICC). Written informed consent was obtained from all patients, and protocols for this study were approved by the Ethics Committee of Jinling Hospital.

### Data acquisition

By consulting the electronic medical record system, information of patients meeting the standard was collected, including preoperative clinical indicators (gender, age, body mass index, smoking and alcohol history, glycosylated hemoglobin (HbAlc), albumin, etc.), intraoperative indicators (operation mode, operation time, operative blood loss, etc.) and postoperative indicators (oral feeding time, oral food tolerance, blood glucose, complications, index of nutrition, immune and inflammation, TNM stage, differentiation degree and clinical outcome, etc.). Index of nutrition, immune and inflammation, weight change, quality of life score and T12-SMA were reviewed 1 month after discharge. Postoperative complications were graded according to the severity grading system (2009, Clavien-Dindo) and quality of life scores were obtained by QLQ-C30 questionnaire.

### Surgery methods

After admission, all patients underwent routine gastroscopy, chest and abdomen CT, electrocardiogram and pulmonary function examination to exclude surgery contraindications. Surgical methods include open surgery and minimally invasive surgery (including thoracoscopic radical esophagectomy and Da Vinci robot-assisted radical esophagectomy).

### Glycemic management

Preoperative blood glucose monitoring was conducted, of which blood glucose was measured 2 h before and after meals each day. The blood glucose value after meals was controlled below 7 ~ 11.1 mmol/L, and the blood glucose was maitained stable for more than 3 days before the operation. After operation, blood glucose was monitored once a day at 6, 9, 11, 14, 17, 20 and 22 o ‘clock. The blood glucose was adjusted by subcutaneous injection of ordinary insulin as to control it smoothly at 7.0 ~ 11.1 mmol/L.

### Postoperative nutritional support

Postoperative nutritional support was provided to patients in both groups according to the ESPEN (the guidelines of the European Society of Parenteral and Enteral Nutrition) and the guidelines of the American Nutrition Association. Patients in both groups received the same nutritional support after surgery. Supplemental Parenteral Nutrition (SPN) was used on the same day after surgery according to the “permissible low-calorie principle” [20-25 kcal/kg]. In addition, 500 ml 5% glucose and sodium chloride injection was pumped through the nasoenteral nutrition tube or jejunal stoma tube 12 h after the operation. According to the patient’s tolerance, enteral nutrition (EN) and perenteral nutrition (PN) preparation were combined with intravenous infusion within 24 h after the operation, with a total daily calories of 30 kcal/kg. Patients began to drink water after anus exhaust defecation, and gradually shifted to liquid food.

### Home nutrition

At discharge, the patients were given a follow-up table of home nutrition and were required to record the patient’s weight, diet and food intake, gastrointestinal reactions and other information every day. Patients received home enteral nutrition through oral nutrition solution or jejunostomy tube every day. On the basis of normal diet of patients, professional dietitians calculated the amount of extra nutrition needed, and conducted dietary guidance by telephone.

### Statistical analysis

Statistical software SPSS 23.0 was used to analyze the data. Quantitative data were presented as mean and standard deviation (Mean ± SD). Independent sample t test was used for inter-group comparison. And chi-square test was used for inter-group comparison. *P* < 0.05 indicated statistically significant difference (*p < 0.05; **p < 0.01; ***p < 0.001).

## Results

### General information

A total of 121 patients were included in this study. According to the median time (7 days) of first postoperative oral feeding time, they were divided into EOF group (67 cases) and LOF group (54 cases). Among them, there were 50 males and 17 females in EOF group, with an average age of (63.37 ± 7.49) years. There were 41 males and 13 females in the LOF group, with an average age of (63.74 ± 7.22) years. The mean duration of diabetes was 4.96 ± 2.56 years in the EOF group and 5.27 ± 2.82 years in the LOF group. There were no statistically significant differences in gender, age and body mass index between the two groups (*p* > 0.05) (Table 1).

**Table 1:** General information of included patients

Characteristics	EOF (*n* = 67)	LOF (*n* = 54)	*p* value
Gender			0.869
Male	50	41	
Female	17	13	
Age (years)	63.37 ± 7.49	63.74 ± 7.22	0.784
BMI (kg /m2)	22.69 ± 2.19	23.12 ± 2.61	0.326
Underlying diseases			0.556
Yes	14	9	
No	53	45	
Smoking history			0.673
Yes	41	31	
No	26	23	
Alcohol history			0.681
Yes	44	36	
No	23	18	
Neoadjuvant chemotherapy			0.828
Yes	7	5	
No	60	49	
Preoprative HbAlc	7.58 ± 1.36	7.91 ± 1.64	0.228
Duration of diabetes history (years)	4.96 ± 2.56	5.27 ± 2.82	0.528
Operation type			0.324
Minimally invasive	49	35	
Open	18	19	
Identical parts			0.625
Cervical	51	41	
Intrathoracic	16	13	
Operating time (min)	208.27 ± 36.82	215.64 ± 41.52	0.303
Intraoperative blood loss (ml)	227.62 ± 50.26	239.81 ± 55.83	0.209
Tumor location			0.284
Upper	4	6	
Middle	45	29	
Lower	18	19	
Number of dissected lymph nodes	18.62 ± 11.16	20.34 ± 9.86	0.377
Degree of tumor differentiation			0.439
Well	9	10	
Moderately	40	26	
Poorly	18	18	
TNM stage			0.307
T1	13	7	
T2	34	24	
T3	20	23	

### Blood glucose levels

**Fig. 1:**
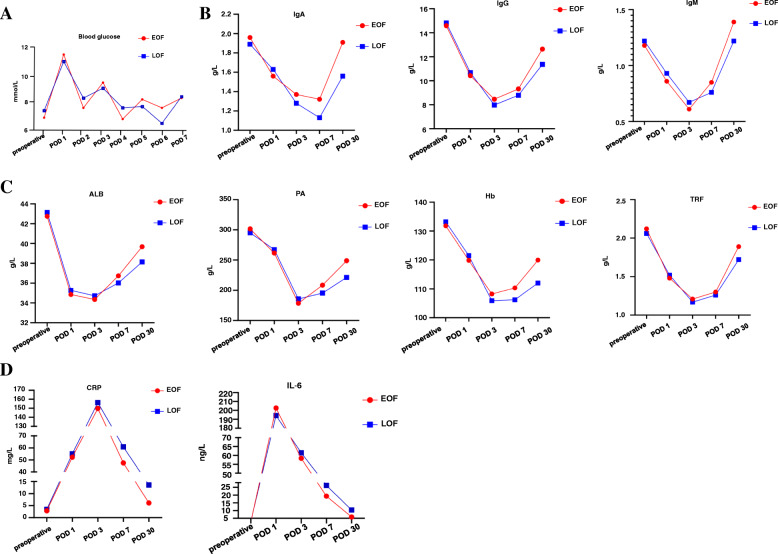
**a** EOF group and LOF group showed similar blood glucose levels after surgery. **b** EOF group showed higher IgA level in POD7 and higher IgA, IgG and IgM level in POD30. **c** EOF group presented higher PA level in POD 7 and higher ALB, PA, TRF and Hb level in POD 30. **d** EOF group had lower CRP and IL-6 level in POD 7 and POD 30

Within 7 days after the operation, the blood glucose levels in EOF group and LOF group fluctuated from 6.86 to 11.47 mmol/L and 6.52 to 10.88 mmol/L, respectively. The blood glucose level varied greatly in the first 2 days after surgery, but both group showed similar blood glucose levels after surgery (*p* > 0.05) (Fig. 1a). It suggested that EOF might not affect the control of blood glucose.

### Immune index

The immune index of the two groups showed a decreasing trend after the operation, with in POD 3 falling to the lowest and then slowly recovering. There was no significant difference on immune index between the two groups in the preoperative day, POD 1 and POD 3 (*p* > 0.05). However, EOF group showed higher IgA (1.32 ± 0.53 VS 1.13 ± 0.41, *p* = 0.032) level in POD7 and higher IgA (1.91 ± 0.78 VS 1.56 ± 0.65, *p* = 0.009), IgG (12.64 ± 2.86 VS 11.36 ± 2.71, *p* = 0.014) and IgM (1.39 ± 0.42 VS 1.22 ± 0.37, *p* = 0.021) level in POD30 (Table 2) (Fig. 1b). These results indicated that EOF was conducive to the restoration of immunity.

**Table 2:** Immune index of the two groups

Immune index	EOF (*n* = 67)	LOF (*n* = 54)	*p* value
Preoperative			
IgA/(g/L)	1.96 ± 0.85	1.89 ± 0.91	0.663
IgG/(g/L)	14.59 ± 3.22	14.81 ± 2.96	0.699
IgM/(g/L)	1.18 ± 0.33	1.22 ± 0.37	0.531
POD 1			
IgA/(g/L)	1.56 ± 0.69	1.63 ± 0.55	0.547
IgG/(g/L)	10.42 ± 2.16	10.68 ± 2.83	0.568
IgM/(g/L)	0.86 ± 0.27	0.93 ± 0.21	0.121
POD 3			
IgA/(g/L)	1.37 ± 0.58	1.28 ± 0.46	0.355
IgG/(g/L)	8.46 ± 2.63	7.98 ± 2.14	0.281
IgM/(g/L)	0.61 ± 0.19	0.67 ± 0.23	0.119
POD 7			
IgA/(g/L)	1.32 ± 0.53	1.13 ± 0.41	0.032*
IgG/(g/L)	9.32 ± 2.13	8.79 ± 2.27	0.189
IgM/(g/L)	0.85 ± 0.22	0.76 ± 0.29	0.055
POD 30			
IgA/(g/L)	1.91 ± 0.78	1.56 ± 0.65	0.009*
IgG/(g/L)	12.64 ± 2.86	11.36 ± 2.71	0.014*
IgM/(g/L)	1.39 ± 0.42	1.22 ± 0.37	0.021*

Note: *--*P* < 0.05

### Nutrition index

Similarly, nutrient level declined sharply after surgery and slowly rebounded in POD3. There was no significant difference in nutrition index between the two groups in the preoperative day, POD 1 and POD 3 (*p* > 0.05). EOF group presented higher PA (208.62 ± 27.12 VS 195.37 ± 32.78, *p* = 0.016) level in POD 7 and higher ALB (39.67 ± 3.16 VS 38.14 ± 3.83, *p* = 0.018), PA (248.96 ± 47.85 VS 211.38 ± 55.23, *p* = 0.004), TRF (1.89 ± 0.42 VS 1.72 ± 0.35, *p* = 0.019) and Hb (119.95 ± 14.02 VS 112.06 ± 18.73, *p* = 0.009) level in POD 30 (Table 3) (Fig. 1c). This given us a hint that EOF could improve the nutritional status of patients after surgery.

**Table 3:** Nutrition index of the two groups

Nutrition index	EOF (*n* = 67)	LOF (*n* = 54)	*p* value
Preoperative			
ALB (g/L)	42.74 ± 4.27	43.15 ± 4.68	0.616
PA (mg/L)	301.62 ± 77.13	295.28 ± 84.32	0.667
TRF (g/L)	2.12 ± 0.36	2.06 ± 0.22	0.285
Hb (g/L)	131.83 ± 19.71	133.21 ± 24.37	0.737
POD 1			
ALB (g/L)	34.86 ± 3.15	35.27 ± 3.72	0.513
PA (mg/L)	261.54 ± 35.64	267.36 ± 43.39	0.423
TRF (g/L)	1.48 ± 0.23	1.52 ± 0.31	0.417
Hb (g/L)	119.86 ± 12.76	121.49 ± 18.43	0.567
POD 3			
ALB (g/L)	34.36 ± 2.64	34.73 ± 2.17	0.409
PA (mg/L)	178.62 ± 22.12	185.73 ± 28.75	0.127
TRF (g/L)	1.21 ± 0.23	1.17 ± 0.11	0.243
Hb (g/L)	108.26 ± 15.64	105.91 ± 12.53	0.372
POD 7			
ALB (g/L)	36.74 ± 3.22	36.02 ± 3.64	0.257
PA (mg/L)	208.62 ± 27.12	195.37 ± 32.78	0.016*
TRF (g/L)	1.30 ± 0.18	1.26 ± 0.11	0.155
Hb (g/L)	110.36 ± 12.34	106.24 ± 18.67	0.148
POD 30			
ALB (g/L)	39.67 ± 3.16	38.14 ± 3.83	0.018*
PA (mg/L)	248.96 ± 47.85	211.38 ± 55.23	0.004*
TRF (g/L)	1.89 ± 0.42	1.72 ± 0.35	0.019*
Hb (g/L)	119.95 ± 14.02	112.06 ± 18.73	0.009*

Note: *--*P* < 0.05

### Inflammatory index

There was no significant difference in inflammatory index between the two groups in the preoperative day, POD 1 and POD 3 (*p* > 0.05). Both CRP and IL-6 increased after surgery, of which CRP reached peak in POD 3 and IL-6 in POD 1. EOF group had lower CRP and IL-6 level in POD 7 (CRP, 47.58 ± 25.72 VS 60.87 ± 30.26, *p* = 0.01; IL-6, 19.34 ± 12.67 VS 26.19 ± 10.73, *p* = 0.002) and POD 30 (CRP, 6.13 ± 13.25 VS 13.57 ± 18.96, *p* = 0.013; IL-6, 5.86 ± 6.34 VS 10.35 ± 5.82, *p* = 0.0001) (Table 4) (Fig. 1d). It revealed that EOF could promote the recovery of inflammatory response.

**Table 4:** Inflammatory index of the two groups

Inflammatory index	EOF (*n* = 67)	LOF (*n* = 54)	*p* value
Preoperative			
CRP (mg/L)	2.86 ± 3.37	3.48 ± 5.72	0.46
IL-6 (ng/L)	2.96 ± 3.17	2.35 ± 3.89	0.344
POD 1			
CRP (mg/L)	52.34 ± 10.24	55.12 ± 13.36	0.198
IL-6 (ng/L)	202.64 ± 95.51	194.12 ± 88.23	0.615
POD 3			
CRP (mg/L)	149.78 ± 57.86	156.31 ± 46.14	0.502
IL-6 (ng/L)	58.36 ± 29.68	61.49 ± 33.72	0.588
POD 7			
CRP (mg/L)	47.58 ± 25.72	60.87 ± 30.26	0.01*
IL-6 (ng/L)	19.34 ± 12.67	26.19 ± 10.73	0.002*
POD 30			
CRP (mg/L)	6.13 ± 13.25	13.57 ± 18.96	0.013*
IL-6 (ng/L)	5.86 ± 6.34	10.35 ± 5.82	0.0001*

Note: *--*P* < 0.05

### Postoperative complications

Total complications in EOF group and LOF group were 25 cases (37.31%) and 21 cases (38.87%), respectively. The incidence of anastomotic fistula, incision infection and pulmonary infection complications in EOF group and LOF group were respectively (8.96% VS 9.26%), (7.46% VS 5.56%) and (7.46% VS 9.26%), and there was no statistically significant difference in the incidence of these three complications between the two groups (*p* > 0.05) (Table 5). This showed that EOF might not increase the incidence of complications.

**Table 5:** Postoperative complications in the 2 groups

Postoperative complications	EOF (*n* = 67)	LOF (*n* = 54)	*p* value
Clavien-Dindo Degree I-II	17	14	0.945
Pulmonary infection	4	4	0.752
Incision infection	5	3	0.675
Gastrointestinal dysfunction	5	4	0.991
Injury of recurrent nerve	3	3	0.786
Clavien-Dindo Degree III-IV	8	7	0.643
Severe pulmonary infection and respiratory failure	1	1	> 0.999
Hydropneumothorax	1	0	1
Anastomotic fistula	6	5	0.954
Anastomotic or thoracic bleeding requiring reoperation	0	1	0.446
Total	25	21	0.859

### Body weight loss and T12-SMA variation

**Fig. 2:**
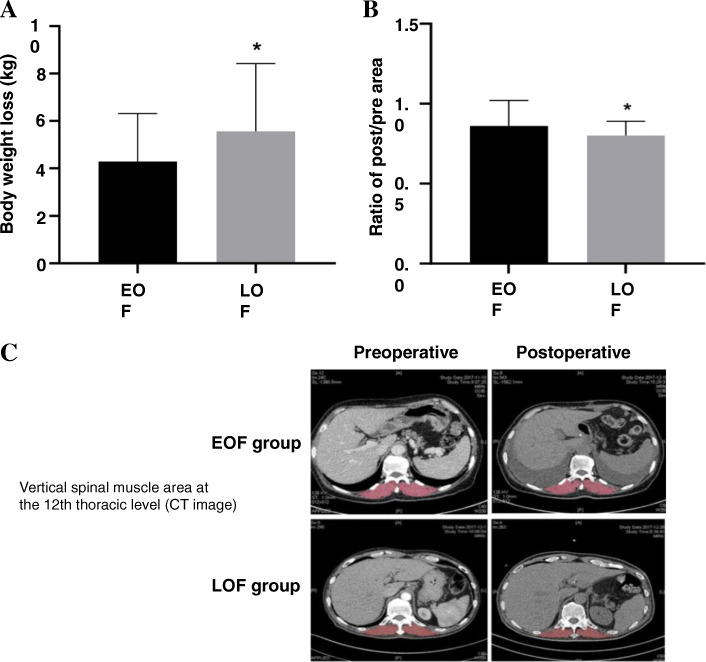
**a** The body weight loss of patients in EOF group was lower than that in LOF group in POD 30. **b** T12-SMA variation was greater in the EOF group. **c** CT images of calculating T12-SMA were shown

The body weight loss of patients in EOF group was lower than that in LOF group in POD 30 (4.23 ± 2.06 VS 5.56 ± 2.86, *p* = 0.004) (Fig. 2a). Moreover, T12-SMA variation (the post/pre ratio) was greater in the EOF group (0.87 ± 0.25 VS 0.79 ± 0.16, *p* = 0.043) (Fig. 2b-c). These results suggested that EOF could inhibited the loss of body weight and skeletal muscle caused by surgery.

### EORTC QLQ-C30 scores

As shown in Table S1, there was no statistical difference in preoperative QLQ-C30 scores between the two groups. However, 30 days after surgery, the overall health of patients in EOF group was better than that in LOF group (61.33 ± 10.18 VS 53.96 ± 14.87, *p* = 0.001). And in the functional rating scale, the EOF group was better than the LOF group in terms of physical function (59.12 ± 17.74 VS 50.35 ± 16.48, *p* = 0.006) and emotional function (61.17 ± 15.73 VS 54.98 ± 12.61, *p* = 0.021). Moreover, on score of symptoms rating scale, fatigue (50.82 ± 19.47 VS 59.16 ± 18.04, *p* = 0.017), loss of appetite (27.67 ± 20.36 VS 38.62 ± 21.68, *p* = 0.005) and diarrhea (22.96 ± 16.08 VS 31.32 ± 17.79, *p* = 0.008) of the EOF group were lower than those of the LOF group (Table 6). These results showed that EOF might reduce the incidence of some certain symptoms and improve the quality life of patients.

**Table 6:** Postoperative QLQ-C30 scores of the two group

Items	EOF (*n* = 67)	LOF (*n* = 54)	*p* value
Functional Rating Scale			
physical function	59.12 ± 17.74	50.35 ± 16.48	0.006*
role function	46.29 ± 15.64	44.84 ± 13.28	0.589
emotional functioning	61.17 ± 15.73	54.98 ± 12.61	0.021*
cognitive function	81.27 ± 10.11	79.66 ± 11.89	0.423
social function	51.62 ± 15.81	49.39 ± 16.76	0.454
Symptoms Rating Scale			
fatigued	50.82 ± 19.47	59.16 ± 18.04	0.017*
nausea and vomiting	22.86 ± 12.76	24.78 ± 16.67	0.474
pain	23.69 ± 15.95	24.86 ± 16.59	0.694
anhelation	18.23 ± 16.95	19.27 ± 18.36	0.747
insomnia	28.66 ± 16.36	30.64 ± 17.39	0.521
appetite loss	27.67 ± 20.36	38.62 ± 21.68	0.005*
constipation	19.26 ± 16.34	17.92 ± 17.72	0.667
diarrhea	22.96 ± 16.08	31.32 ± 17.79	0.008*
financial difficulty	31.82 ± 24.57	30.67 ± 22.09	0.79
General health status	61.33 ± 10.18	53.96 ± 14.87	0.001*

Note: *--*P* < 0.05

## Discussion

Esophagectomy is the predominant treatment for patients with esophageal cancer at present. Due to the complexity, large trauma and long duration of the surgery, patients are at a state of stress and have a high incidence of postoperative complications, including anastomotic fistula, pulmonary infection, respiratory failure, etc. [14]. Patients with esophageal cancer usually have malnutrition for its difficult swallowing and tumor consumption. And for patients complicated with type II diabetes, the blood glucose fluctuates widely and stress response is violent, thus increasing the inflammatory reaction, impairing the immune system and decreasing tissue repair ability [15]. Therefore, esophageal cancer patients complicated with type II diabetes are more likely to suffer postoperative complications and tend to have a poorer life quality [16, 17]. It is of great importance to reduce the surgical risk and improve the prognosis of these group of patients.

With the development of the concept of Enhanced Recovery After Surgery (ERAS) and its application in the field of surgery, patients can better endure surgical stress and recover more quickly. The core theory of ERAS is to allow patients to return to the physiological state, to relieve the body’s stress state, and to accelerate the early recovery of patients as soon as possible after surgery [18, 19]. In the traditional surgical treatment scheme for esophageal cancer, clinicians often worry that the early postoperative oral feeding may increase the incidence of anastomotic leakage. Hence, patients need to perform routine gastrointestinal decompression and fasting for 5–7 days after surgery [20]. For patients complicated with diabetes, anastomotic healing tends to be delayed, so postoperative fasting time of patients is usually prolonged in clinical practice [21]. However, in the treatment mode of ERAS, early oral feeding is the most physiological way of nutrition delivery, which has been regarded as one of the most crucial measures [22]. Incresing evidences have shown that early oral feeding after digestive tract surgery was safe and feasible, which did not increase the incidence of postoperative complications such as anastomotic fistula and pulmonary infection, and meanwhile benefited patients for their long-term quality of life [23–25]. At present, there are few reports on the safety and benefit of early oral feeding in patients of esophageal cancer complicated with type II diabetes.

Patients with esophageal cancer are often accompanied by malnutrition due to different degrees of eating obstruction and chronic consumption, and the high catabolism caused by surgical trauma and post-operative stress reaction will further aggravate malnutrition and immunosuppression [26]. In this study, we used ALB, PA, TRF, Hb to evaluate the nutrition status and IgA, IgG, IgM to measre the immune status, and results revealed that postoperative nutritional and immune indexes of patients in EOF group were higher than those in LOF group, especially in POD 7 and POD 30. And inflammatory response is one of the main manifestations of postoperative. Variations in serum inflammatory cytokines can objectively reflect the state of postoperative inflammatory response of patients [27]. The CRP and IL-6 levels in EOF group were lower than LOF in POD7 and POD30, which suggested that early oral feeding led the inflammatory response subside faster. Furthermore, variations in digestive tract structure and dietary habits after surgery result severe weight loss of severe skeletal muscle [28]. The body weight loss and T12-SMA variation were less in EOF group. Lastly, QLQ-C30 scores of the EOF group were also higer than those in LOF group. Previous studies have proved that early oral feeding can protect the intestinal mucosal barrier of patients undergoing gastrointestinal surgery, thus to improve nutrition, immunity, and promote the recovery of intestinal and organ functions [29]. And our results suggested that early oral feeding was a safe intervention for patients of esophageal cancer complicated with type II diabetes.

## Conclusion

In summary, our study retrospectively analyzed the clinical data of 121 patients to explore the safety of early oral feeding in patients of esophageal cancer complicated with type II diabetes. And results showed that early oral feeding could speed up the recovery of nutritional and immune status, decrease the inflammatory response and weight loss of surgery, and meanwhile do not increase the incidence of complications.

### Weakness and expectation

This study has some limitations. First, the patient data in this study was collected from a single institution database. Therefore, there is a certain degree of selection bias. Second, it includes only a retrospective study, and intrinsic errors and deviations in its design may inevitably affect the results. This study requires further multi-center, large sample, and prospective studies to verify our results. Our research group has continued to collect the number of relevant cases, and the next step is to cooperate with other centers for research.

## Supplementary Information

**Additional file 1: Table S1.** Preoperative QLQ-C30 scores of the two group.

## Data Availability

Not applicable.

## Abbreviations

EOF: Early oral feeding
LOF: Late oral feeding
QLQ: Quality of Life Questionnaire
ALB: Albumin
PA: Prealbumin
TRF: Transferrin
Hb: Hemoglobin
CRP: C-Reactive Protein
IL-6: Interleukin-6
HbAlc: Glycosylated hemoglobin
EN: Enteral Nutrition
PN: Enteral Nutrition
POD: Postoperative Day
ERAS: Enhanced Recovery After Surgery
